# Predictors of healthcare professionals' intention and behaviour to encourage physical activity in patients with cardiovascular risk factors

**DOI:** 10.1186/1471-2458-11-246

**Published:** 2011-04-19

**Authors:** Barbara Sassen, Gerjo Kok, Luc Vanhees

**Affiliations:** 1 Department of Health and Lifestyle, University of Applied Sciences, Utrecht, the Netherlands; 2 Department of Work and Social Psychology, Maastricht University, Maastricht, the Netherlands; 3 Department of Rehabilitation Sciences, KU Leuven, Leuven, Belgium

## Abstract

**Background:**

Healthcare professionals can play a crucial role in optimizing the health status of patients with cardiovascular risk factors (abdominal obesity, high blood pressure, low HDL cholesterol, elevated triglycerides and elevated blood glucose). In order to do this, it is imperative that we understand the social-cognitive determinants (including habits) that underlie healthcare professionals' intention and the corresponding behavior of actually encouraging patients with cardiovascular risk factors to engage in physical activity.

**Methods:**

In this longitudinal Professionals' Intention and Behavior (PIB) study, healthcare professionals (N = 278, aged 20-61 years with approximately 60% having attained an education level exceeding bachelor's degree, types of healthcare professionals 60% in physiotherapy and 40% in nursing) completed online surveys measuring the social-cognitive determinants of healthcare professionals' intention and the corresponding behavior of actually encouraging patients with cardiovascular risk factors to engage in physical activity.

**Results:**

Social-cognitive determinants accounted for 41% (p < .001) of the variance in healthcare professionals' intention to encourage physical activity among cardiovascular patients. Important correlates of intention were attitude (β = .443, p < .001), subjective norms (β = .201, p < .001) and perceived behavioral control (β = .137, p < .01). With respect to the self-reported behavior of encouraging patients, social-cognitive determinants accounted for 29% (p < .001) of the variance. Intentions (β = .311 p < .001), habit (β = .163 p < .01), and barriers (β = -.239 p < .001) were significant correlates of professionals' behavior of encouraging patients to engage in physical activity.

We explored the congruence between healthcare professionals' intention to encourage patients and the self-reported behavior of encouraging patients. We found that intention and behavior were congruent in 39.7% of the healthcare professionals. Additionally, the intention to encourage and the corresponding behavior of encouraging was incongruent in 31.7% of the healthcare professionals.

**Conclusions:**

In the prevention of cardiovascular disease, healthcare professionals' intention to encourage physical activity among patients and subsequent behavior of encouraging patients is important for the improvement of patients' cardiovascular risk profiles. We found that the intentions and self-reported behavior of healthcare professionals working with patients with cardiovascular risk factors can be predicted by social-cognitive determinants thus implying that efforts to change and strengthen the intention-behavior relationship of healthcare professionals may have beneficial effects for cardiovascular risk patients (Trial ID: ECP-92).

## Background

Healthcare professionals can play an essential role in optimizing the health status of patients with cardiovascular risk factors. However, a substantial gap between clinical research findings and the daily practice of healthcare professionals remains [1-7]. Though nowadays it is common to work evidence-based for healthcare professionals, the daily practice for healthcare professionals is sometimes different. In order to increase congruence between theory and practice and better support patients, it is imperative that we gain an improved understanding of healthcare professionals' behavior and the determinants that underlie their behavior.

Cardiovascular risk factors, namely abdominal obesity, high blood pressure, low HDL cholesterol (HDL-C), elevated triglycerides and elevated blood glucose levels, increase one's risk for cardiovascular disease, type 2 diabetes and all-cause mortality [8-11]. According to the classification based on the extended ATPIII criteria put forth by the National Cholesterol Education Program's Adult Treatment Panel III definition, people at cardiovascular risk are having one or more of the following risk factors: abdominal obesity (for men a waist circumference >102 cm and for women >88 cm); high blood pressure (with a systolic blood pressure of ≥130 mm Hg, or diastolic ≥90 mmHg, or being on antihypertensive drug treatment); people with low HDL cholesterol (high-density lipoprotein, for men <1.03 mmol/L, for women <1.30 mmol/L) or being on medication; elevated triglycerides (≥1.70 mmol/L, or being on medication that reduces triglycerides, and; elevated blood glucose (≥6.1 mmol/L, or being on medication that reduces blood glucose [8,9].

The number of patients with cardiovascular risk factors is increasing and the prevalence is being exacerbated by decreasing physical activity levels [9,10,12]. Research has demonstrated that physical activity and physical fitness have a preventive effect on cardiovascular morbidity and mortality [13-17]. Physical activity and physical fitness are associated with important health benefits for every cardiovascular risk factor and also for the group of interrelated risk factors. Physical activity and physical fitness can have a beneficial effect on lipids, blood pressure, glucose metabolism, and on body composition [9,11,18-27]. In a recent study on physical activity, we found that particularly (high) intensity physical activity impacts cardiovascular risk factors and concluded that interventions should focus on high intensity physical activity leading to physical fitness [28].

In order to enable the development of effective cardiovascular disease prevention interventions, we must understand how healthcare professionals can best be engaged to promote an active lifestyle in cardiovascular patients. Consequently, in this study, we investigated the social-cognitive determinants of healthcare professionals' intention to encourage physical activity among cardiovascular patients as posited by the Theory of Planned Behavior and other relevant social psychological theories [29-38]. The Theory of Planned Behavior is considered appropriate for explaining both healthcare professionals' intentions and behavior [1,5,30].

According to the Theory of Planned Behavior, healthcare professionals' behavior of encouraging physical activity among cardiovascular patients is predicted by their intention to encourage physical activity among these patients and behavior is also determined by habits and barriers. Behavioral intention can be explained by three social-cognitive determinants, namely attitude, subjective norm, and perceived behavioral control [38]. Attitude refers to the general evaluation of the behavior, and is determined by behavioral beliefs (perceptions regarding the advantages and disadvantages of the behavior) and perceptions regarding the consequences of the behavior. The subjective norm refers to the perceived social approval for the behavior, and is determined by expectations regarding whether important reference individuals or groups will approve of the behavior. In addition to the subjective norm, the descriptive norm from Bandura's Social Cognitive Theory is an extension of the Theory of Planned Behavior [39]. The descriptive norm can be considered as the behavior of others in the social environment. The moral norm, also an extension of the Theory of Planned Behavior, is essential as the individuals' responsibility and the individuals' moral obligation towards the behavior to act in a specific way [30]. Perceived behavioral control is one's confidence in one's ability to perform a specific behavior and is determined by control beliefs that are based on perceptions of opportunities as well as perceived barriers and required resources [38].

People intend to engage in behaviors when the behavior is evaluated as positive, when one thinks that significant others find the behavior to be important, and when the behavior is considered to be under personal control. Behavior can be predicted by intention, barriers and habit [33]. Habit, as an extension of the Theory of Planned Behavior, can influence the intention-behavior relationship. Habit is determined by past behavior and concerns the individuals' personal experience with a specific behavior in a stable context, such as a healthcare setting [40]. Barriers can obstruct the behavior, even though the intention to engage in the behavior is positive. According to the Theory of Planned Behavior, the influence of general dispositions and socio-demographic factors are mediated by attitude, subjective norms and perceived behavioral control.

In order to make cardiovascular prevention and the implementation of interventions directed at optimizing the health status of cardiovascular patients effective, it is imperative that we gain a better understanding of healthcare professionals' behavior and intentions, and the social-cognitive determinants of behavior and intentions. The aims of this study were first to explore the relative importance of social-cognitive variables in relation to healthcare professionals' intention and behavior. Secondly we investigated the congruence between healthcare professionals' intention to encourage physical activity in cardiovascular risk patients and the self-reported behavior of encouraging patients.

## Methods

In the Professionals' Intention and Behavior (PIB) study, healthcare professionals completed online surveys measuring their intention to encourage physical activity among cardiovascular risk patients, their self-reported behavior of encouraging cardiovascular risk patients, the social-cognitive determinants of their intentions and behavior, and demographic variables. Participants were recruited from the Department of Physiotherapy and Department of Nursing at the University of Applied Sciences in the Netherlands. Participants were (former) students of the University of Applied Sciences in the Netherlands. Healthcare professionals with at least a Bachelor's degree in nursing (approximately 40% of the study group) or physiotherapy (60% of the study group) and who conduct consultations with cardiovascular risk patients were included in the analyses. In total 739 were invited to complete the online survey, with three reminders send. 572 healthcare professionals completed the survey at time 1 (T1, April 2009) and of those, 278 professionals completed the survey at time 2 (T2, October 2009). Statistical analysis in separated groups revealed no differences between healthcare professionals' with a background in physiotherapy or nursing. The ethics committee of the Maastricht University, The Netherlands, granted approval to the study (Trial ID: ECP-92).

### Assessment of social-cognitive determinants

The survey content was derived from a literature review and in-depth interviews with professionals on how they encourage cardiovascular patients to engage in and maintain physical activity. There were eight elicitation interviews held, four with healthcare professionals with a background in physiotherapy and four in nursing. Four of those professionals were observed in their professional activities for a regular working day. For measuring social-cognitive determinants, no valid questionnaires are available, but only valid procedures. The construction of the questionnaire is, according to the Theory of Planned Behavior, specific to the definition of the behavior and the specification of the research population [33,41]. The questionnaire was piloted, as a result no revisions were made. We collected data at time 1 (T1) and time 2 (T2). At T1 the baseline measurement, we measured all social-cognitive determinants except self-reported behavior. At T1 all social-cognitive determinants underlying behavior were investigated. At T2 the follow-up measure, we measured self-reported behavior. By measuring social-cognitive determinants at T1 (social-cognitive determinants) followed by T2 (behavior) makes it possible to predict behavior from intention.

*Behavior *was assessed by averaging two items: 'Do you encourage cardiovascular patients to become physically active?'. And 'In the past month, how many of your cardiovascular patients did you encourage to become physically active?'. Answers ranged from 'none' (1) to 'all' (7) on a seven-point scale (Cronbach's α = .64). Behavior was also dichotomized as encouragement versus no encouragement. For dichotomizing the social-cognitive determinant behavior, we calculated quintiles of the determinant score. The highest two quintiles were positioned as encouraging behavior and the lowest two quintiles were positioned as less encouragement. We left the 20% in between the two groups (encouraging vs. less encouraging) out, to make a clear distinction between groups.

*Intention *was indexed with three questions: 'Do you intend to encourage cardiovascular patients to become physically active tomorrow and the day after tomorrow?', 'Do you expect to encourage cardiovascular to become physically active tomorrow and the day after tomorrow?', and 'Of the first 10 cardiovascular patients you see, how many do you intend to encourage to become physically active?' (Cronbach's α = .82). Answers ranged on a seven-point scale (1 = 'definitely not' to 7 = 'most definitely'), except for the question 'Of the first 10 cardiovascular patients ...'. We recalculated this question on a 10-point scale multiplying it by 0.7. Intention was also dichotomized as high versus low intention. For dichotomizing the social-cognitive determinant intention, we calculated quintiles of the determinant score. The highest two quintiles were positioned as having a high intention and the lowest two quintiles were positioned as having a low intention. We left the 20% in between the two groups (high vs. low intention) out, to make a clear distinction between groups.

*Attitude *was assessed by ten items on a seven-point scale. Answers ranged on a seven-point scale (1 = bad/very useless to 7 = good/very useful). First we asked 'In my view, encouraging cardiovascular patients to become physically active is very good - very bad' and 'Encouraging cardiovascular patients is very useful - very useless'. Then we asked, 'Is it useful to: assess patients' motivation, assess the pros and cons of physical activity, teach patients how to resist social pressure, teach patients specific skills pertaining to physical activity, teach patients how to handle barriers in regard physical activity, formulate physical activity goals together with patients, teach patients how to handle relapses, and help patients understand the relationship between the specific health problem and physical inactivity?'. These eight items ('Is it useful to ...') were averaged and that score was averaged with the first two item scores to represent attitude (Cronbach's α = .63).

*Perceived behavioral control *was assessed by 11 questions on a seven-point scale. First we asked, 'Do you think that you have the skills and knowledge to encourage cardiovascular patients to become physically active?'. We then asked: 'Do you think you can rely on your skills and knowledge to encourage cardiovascular patients to become physically active?'. Answers ranged from 'no, certainly not' (1) to 'yes, certainly' (7) on a seven-point scale. Thirdly we asked 'Encouraging every cardiovascular patients to become physically active is very difficult (1) - very easy (7)'. Perceived behavioral control was further assessed by an eight item scale that paralleled the eight item scale used for attitudes. 'It is very difficult (1) - very easy (7) to: assess patients' motivation, assess the pros and cons of physical activity, teach patients how to resist social pressure, teach patients specific skills pertaining to physical activity, teach patients how to handle barriers in regard physical activity, formulate physical activity goals together with patients, teach patients how to handle relapses, and help patients understand the relationship between the specific health problem and physical inactivity?'. Once again this scale score was calculated and combined with the previous three items as a measure of perceived behavioral control (Cronbach's α = .78).

*Subjective norm *was measured by four items: 'Most colleagues who are important to me think I should encourage cardiovascular patients to become physically active', 'Most colleagues value that I encourage cardiovascular patients to become physically active', 'Patients value that I encourage them to become physically active', and 'The organization I work for values that I encourage cardiovascular patients to become physically active'. Answers ranged from 'no, certainly not' (1) to 'yes, certainly' (7) on a seven-point scale (Cronbach's α = .73).

*Descriptive norm *was indexed by four items: 'Do you encourage more cardiovascular patients to engage in physical activity than your colleagues do?', 'Do you encourage more cardiovascular patients to engage in physical activity than professionals that have the same academic background as you do?', 'Most colleagues encourage patients to engage in physical activity themselves', and 'Do you consult colleagues when you experience problems encouraging cardiovascular patients to become physically active?'. Answers ranged from 'no, certainly not' (1) to 'yes, certainly' (7) on a seven-point scale (Cronbach's α = .61).

*Moral norm *was assessed by three questions: 'Encouraging patients to engage in physical activity is my professional duty', 'Encouraging patients to engage in physical activity is a moral obligation', and 'Encouraging patients to engage in physical activity is an obvious part of my job'. Responses were provided on a seven-point scale ranging from 'definitely not' (1) to 'most definitely' (7) (Cronbach's α = .76).

*Habit *was measured by two questions [40]: 'Encouraging patients to be physically active is something I do without thinking', and 'Encouraging patients to be physically active is something I do automatically'. Answers ranged on a seven-point scale (1 = 'definitely not' to 7 = 'most definitely') (Cronbach's α = .75).

*Barriers *were indexed by two questions that focused on encouraging patients 'even when one is busy' and 'even when one's organization makes it difficult to encourage patients'. Answers ranged from 'no, certainly not' (7) to 'yes, certainly' (1) on a seven-point scale (Cronbach's α = .69). In this way, we measure perceived barriers, which may be part of perceived behavioral control. Because barriers are relevant in an organizational context, we measured them separately.

A determinant score was calculated for every social-cognitive determinant. Per social-cognitive determinant we summed up the scores on the questions and divided this by the number of items.

### Statistical analysis

Descriptive statistics were first calculated and Chi-square analysis was used to explore the population under study. Subsequently, a correlation matrix of all social-cognitive determinants was generated. To explore the congruence between professionals having a high or low intention and encouraging or no encouraging behavior, Chi-square analyses were conducted on dichotomized variables. Hierarchical regression analyses were then applied to model healthcare professionals' intention and behavior. To do this, we analyzed the association between: 1) intention at T1 and the social-cognitive determinants; entering attitude, perceived behavior control and subjective norm in the first block, and entering descriptive norms and moral norms in the second block of hierarchical regression. 2) behavior at T2, and intention at T1 together with the social-cognitive determinants; entering intention in the first block and habit and barriers in the second block of hierarchical regression. Significance was set at p < .05. Statistical analyses were undertaken with SPSS version 17.0 (SPSS inc. 2009).

## Results

Table 1 presents descriptive statistics for the study population. We found that 73.7% of the healthcare professionals were female. The mean age was 36.2 years (±10.1). In the sample, about 40% completed a Bachelor's degree in nursing or physiotherapy and about 60% were studying at a level above Bachelor's degree or had completed an additional degree after their Bachelor's degree. Statistical analysis in separated groups revealed no differences between healthcare professionals' with a background in physiotherapy or nursing (p < 0.05). Healthcare professionals had 8.58 (±7.70) years experience working as a professional. They reported to have at least 3.69 (±4.23) consultations with one patient. The average number of consultations was 10.1 (±12.2) with one patient. Also, participants indicated that 60.4% (±25.2) of their consultation time is spent on health education.

**Table 1:** Characteristics of the study population

*N *= 278	
Male	26.3% (73)
Female	73.7% (205)
Age, years	36.2 ± 10.1
Professional experience, years	8.58 ± 7.70
Completed Bachelor's degree in nursing or physiotherapy	37.8% (105)
Completed or currently acquiring a degree above Bachelor's level	31.3% (87)
Completed or currently acquiring a Master's degree in nursing or physiotherapy	30.9% (86)
Consultation time devoted to health education	60.4%
Average number of consultations with one patient	10.1 ± 12.2
Minimum number of consultations with one patient	3.69 ± 4.23

Values are percentages (and numbers) or mean ± SD

The correlation matrix (table 2) shows positive correlations between healthcare professionals' intention to encourage physical activity among cardiovascular patients and behavior (a moderate to strong association, *r *= .44). According to the Theory of Planned Behavior, healthcare professionals' intention is determined by their attitude, the subjective norm and their perceived behavioral control in addition to moral and descriptive norms. Positive correlations were found between intention and attitude (a strong association *r *= .59), subjective norm (a moderate-to-strong association, *r *= .45), perceived behavioral control (a moderate-to-strong association, *r *= .41), descriptive norm (a weak-to-moderate association, *r *= .25), and moral norm (a weak association, *r *= .14). Healthcare professionals' behavior was found to be positively related to perceived behavioral control (a weak-to-moderate association, *r *= .28) and the habit of encouraging patients to engage in physical activity (a moderate association *r *= .30). As expected, a moderate-to-strong negative association between barriers and self-reported behavior was found (*r *= -.40).

**Table 2:** Correlations between behavior, intention and social-cognitive determinants

*N *= 278	BehT2	IntT1	Att	PBC	SN	DN	MN	Hab	Barr
Behavior Time 2	BehT2								
Intention Time 1	.44***	IntT1							
Attitude	.27***	.59***	Att						
Perceived behavioral control	.28***	.41***	.43***	PBC					
Subjective norms	.25***	.45***	.43***	.43***	SN				
Descriptive norms	.29***	.25***	.25***	.34***	.50***	DN			
Moral norms	ns	.14*	ns	ns	ns	ns	MN		
Habit	.30***	.27***	.16**	.40***	.19**	.21**	ns	Hab	
Barriers	-.40***	-.38***	-.39***	-.42***	-.40***	-.33***	ns	-.37***	Barr
Mean	5.77	6.20	6.18	5.04	5.63	4.86	5.57	5.29	2.64
SD	0.91	0.94	0.60	0.60	0.77	0.72	1.13	1.26	1.00
Range	1-7	1-7	1-7	1-7	1-7	1-7	1-7	1-7	1-7

Values represent correlation coefficients.

*** p < 0.001, ** p < 0.01, * p < 0.05.

### Congruence between healthcare professionals' intention and behavior

Analyses were undertaken to explore the congruence between the healthcare professionals' intention to encourage physical activity among cardiovascular patients and the self-reported behavior of encouraging patients (table 3). For behavior, we found that 56.8% of the healthcare professionals encourage physical activity among cardiovascular patients. For intention, we found that 51.3% of the healthcare professionals reported positive intentions to encourage physical activity among cardiovascular patients. In terms of congruence between intention and behavior, 39.7% of the healthcare professionals reported both positive intentions and the corresponding behavior of encouraging physical activity among cardiovascular patients. 31.7% of the professionals scored low on intention and had the corresponding behavior of not encouraging patients to engage in physical activity. In addition, 17.1% of the healthcare professionals demonstrated behavior that encourages physical activity among cardiovascular patients despite low intention. Also, 11.5% of the professionals had high scores on intention but failed to perform the self-reported behavior of encouraging physical activity among cardiovascular patients.

**Table 3:** High and low intention at T1 versus the presence or absence of behavior that encourages physical activity in cardiovascular patients at T2

*N = 199*	Behavior T2		
	**Encouragement**	**No encouragement**	**Total**

Intention high T1	69.9 (79) *39.7*	26.7 (23) *11.5*	51.3 (102)
Intention low T1	30.1 (34) *17.1*	73.3 (63) *31.7*	48.7 (97)
Total	56.8 (113)	43.2 (86)	

Chi-square-analysis, p < 0.05;

Values represent percentages and (numbers); italics represent the percentage of the total group.

Healthcare professionals with high versus low intention differed on all social-cognitive determinants (p < .05). Also, healthcare professionals that engaged in the behavior of encouraging physical activity among cardiovascular patients differed significantly from those who did not on all social-cognitive determinants except on moral norm (p < .05) (see table 4).

**Table 4:** Overview scale: means for all items according to high versus low intention and presence versus absence of behavior encouraging physical activity in cardiovascular patients

	Item	Intention T1		Behavior T2	
		Low	High	No encouragement	Encouragement
I	Behavior	5.35 ± 0.93	6.23 ± .0.69***	4.92 ± 0.68	6.42 ± .0.36***
	1 Do you encourage cardiovascular patients to become physically active?	5.75 ± 0.98	6.63 ± 0.64***	5.39 ± 0.89	6.74 ± 0.44***
	2 In the past month, how many of your cardiovascular patients did you encourage to become physically active? (none-all)	5.00 ± 1.24	5.87 ± 0.97***	4.45 ± 1.10	6.12 ± 0.57***
II	Intention	5.21 ± 0.78	6.97 ± 0.07***	5.76 ± 0.99	6.52 ± 0.70***
	1 Do you intend to encourage cardiovascular patients to become physically active tomorrow and the day after tomorrow?	5.34 ± 0.92	7.00 ± 0.00***	5.83 ± 1.06	6.56 ± 0.84***
	2 Do you expect to encourage cardiovascular patients to become physically active tomorrow and the day after tomorrow?	5.22 ± 1.00	7.00 ± 0.00***	5.79 ± 1.21	6.46 ± 0.85***
	3 Of the first 10 cardiovascular patients you see, how many do you intend to encourage to become physically active?	5.08 ± 1.39	6.92 ± 0.22***	5.63 ± 1.47	6.55 ± 0.85***
III	Attitude	5.81 ± 0.62	6.48 ± 0.43***	6.06 ± 0.62	6.27 ± 0.56**
	1 Encouraging cardiovascular patients is very useful - very useless	5.70 ± 1.07	6.68 ± 0.68***	6.08 ± 0.97	6.38 ± 0.96*
	2 In my view, encouraging cardiovascular patients to become physically active is very good - very bad	6.17 ± 0.72	6.68 ± 0.58***	6.33 ± 0.71	6.54 ± 0.67*
	3 Is it useful to explain the relationship between the specific health problem and physical inactivity? (very useful-not useful at all)	6.07 ± 0.86	6.57 ± 0.78***	6.21 ± 0.80	6.41 ± 0.94
	Is it useful to assess patients' motivation to become physically active?	5.87 ± 1.03	6.24 ± 1.05*	6.07 ± 0.96	6.05 ± 1.17
	Is it useful to assess the pros and cons of physical activity?	5.40 ± 1.05	5.89 ± 1.08**	5.65 ± 1.07	5.64 ± 1.22
	Is it useful to teach patients how to resist social pressure regarding physical activity?	5.10 ± 1.28	5.74 ± 0.89***	5.37 ± 1.16	5.46 ± 1.06
	Is it useful to teach patients specific skills pertaining to physical activity?	5.28 ± 1.15	6.11 ± 0.90***	5.46 ± 1.11	5.90 ± 1.10**
	Is it useful to teach patients how to handle barriers in regard physical activity?	5.46 ± 1.11	6.02 ± 0.87***	5.77 ± 0.99	5.74 ± 1.16
	Is it useful to formulate physical activity goals together with patients?	5.73 ± 1.11	6.17 ± 0.95**	5.92 ± 0.99	5.96 ± 1.14
	Is it useful to teach patients how to handle relapses in physical activity?	5.66 ± 0.93	6.13 ± 0.88***	5.79 ± 0.97	6.04 ± 0.85*
IV	Perceived behavioral control	4.76 ± 0.56	5.36 ± 0.53***	4.89 ± 0.57	5.15 ± 0.57***
	1 Do you think that you have the skills and knowledge to encourage cardiovascular patients to become physically active?	5.35 ± 1.00	6.18 ± 0.84***	5.50 ± 0.97	5.93 ± 0.93**
	2 Do you think you can rely on your skills and knowledge to encourage cardiovascular patients to become physically active?	5.52 ± 1.05	6.39 ± 0.74***	5.68 ± 0.92	6.09 ± 0.98**
	3 Encouraging every cardiovascular patient to become physically active is very difficult-very easy	3.83 ± 1.18	4.37 ± 1.22**	3.96 ± 1.15	4.12 ± 1.24
	4 It is very easy-very difficult to explain the relationship between the specific health problem and physical inactivity	5.27 ± 1.03	5.59 ± 1.00*	5.22 ± 0.99	5.55 ± 0.95**
	It is very easy-very difficult to assess patients' motivation to become physically active.	4.30 ± 1.19	4.79 ± 1.14**	4.45 ± 1.10	4.54 ± 1.19
	It is very easy-very difficult to assess the pros and cons of physical activity.	4.66 ± 1.04	5.04 ± 1.05**	4.84 ± 0.94	4.79 ± 1.06
	It is very easy-very difficult to teach patients how to resist social pressure regarding physical activity.	3.67 ± 1.16	3.96 ± 1.20	3.73 ± 1.11	3.76 ± 1.16
	It is very easy-very difficult to teach patients specific skills pertaining to physical activity.	4.69 ± 1.12	4.97 ± 1.12	4.71 ± 1.09	4.88 ± 1.10
	It is very easy-very difficult to teach patients how to handle barriers to physical activity.	4.06 ± 1.12	4.37 ± 1.09*	3.95 ± 1.04	4.33 ± 1.06**
	It is very easy-very difficult to formulate physical activity goals together with patients.	4.93 ± 0.93	5.08 ± 1.07	4.90 ± 0.99	5.00 ± 1.04
	It is very easy-very difficult teach patients how to handle relapses in physical activity.	3.97 ± 1.08	4.18 ± 1.22	3.93 ± 1.15	4.03 ± 1.10
V	Subjective norms	5.28 ± 0.73	5.95 ± 0.65***	5.43 ± 0.78	5.77 ± 0.73***
	1 Most colleagues who are important to me think I should encourage cardiovascular patients to become physically active.	4.97 ± 1.31	5.82 ± 1.37***	5.20 ± 1.22	5.56 ± 1.51*
	2 Most colleagues value that I encourage cardiovascular patients to become physically active.	5.45 ± 0.87	6.18 ± 0.99***	5.65 ± 0.95	5.99 ± 1.00**
	3 Patients value that I encourage them to become physically active.	4.99 ± 0.90	5.32 ± 0.98*	5.04 ± 0.99	5.27 ± 0.90
	4 The organization I work for values that I encourage cardiovascular patients to become physically active.	5.71 ± 1.08	6.51 ± 0.77***	5.86 ± 1.08	6.30 ± 0.93**
VI	Descriptive norms	4.66 ± 0.71	5.04 ± 0.67***	4.70 ± 0.74	5.00 ± 0.66**
	1 Do you encourage more cardiovascular patients to engage in physical activity than your colleagues do?	4.32 ± 0.99	4.75 ± 1.13**	4.42 ± 0.99	4.66 ± 1.09
	2 Do you encourage more cardiovascular patients to engage in physical activity than professionals that have the same academic background as you do?	4.35 ± 0.67	4.71 ± 0.73***	4.41 ± 0.70	4.66 ± 0.74**
	3 Most colleagues encourage patients to engage in physical activity themselves.	5.45 ± 1.16	5.93 ± 1.05**	5.51 ± 1.15	5.86 ± 1.12*
	4 Do you consult colleagues when you experience problems encouraging cardiovascular patients to become physically active?	4.53 ± 1.48	4.82 ± 1.32	4.50 ± 1.42	4.83 ± 1.37
VII	Moral norm	5.41 ± 0.87	5.75 ± 1.25*	5.46 ± 0.94	5.62 ± 1.24
	1 Encouraging patients to engage in physical activity is my professional duty.	5.61 ± 1.04	6.12 ± 1.31**	5.72 ± 1.02	5.87 ± 1.44
	2 Encouraging patients to engage in physical activity is a moral obligation.	4.77 ± 1.45	5.11 ± 1.60	4.85 ± 1.39	4.99 ± 1.61
	3 Encouraging patients to engage in physical activity is an obvious part of my job.	5.87 ± 0.88	6.03 ± 1.59	5.81 ± 1.18	6.02 ± 1.38
VIII	Habit	4.90 ± 1.21	5.64 ± 1.15***	4.87 ± 1.35	5.51 ± 1.10***
	1 Encouraging patients to be physically active is something I do without thinking.	4.57 ± 1.60	5.44 ± 1.58***	4.55 ± 1.67	5.25 ± 1.55**
	2 Encouraging patients to be physically active is something I do automatically	5.25 ± 1.11	5.86 ± 1.02***	5.21 ± 1.34	5.79 ± 0.94***
IX	Barriers	3.05 ± 0.89	2.24 ± 0.99***	2.96 ± 0.87	2.43 ± 0.99***
	1 I encourage patients to become physically active even when I am busy (no, certainly not-yes, certainly).	2.74 ± 1.17	2.02 ± 1.10***	2.69 ± 1.13	2.12 ± 1.12***
	2 I encourage patients to become physically active even when my organization makes it difficult to encourage patients.	3.31 ± 1.13	2.46 ± 1.24***	3.16 ± 1.08	2.69 ± 1.24**

*** p < .001, ** p < .01, * p < .05.

### Intention and behavior predicted by social-cognitive variables

Regression analyses identified correlates of healthcare professionals' intention to encourage physical activity among cardiovascular patients and the behavior of encouraging these patients (table 5). Social-cognitive variables accounted for 41% (p < .001) of the variance in intention. Intention was, in turn, predicted by attitude (β = .443, p < .001), subjective norms (β = .201, p < .001), and perceived behavioral control (β = .137, p < .05). With respect to the self-reported behavior of encouraging physical activity among cardiovascular patients, 29% of the variance (p < .001) was explained by intention (β = .311, p < .001), next to habit (β = .163, p < .01) and barriers (β = -239, p < .001). With respect to the self-reported behavior, intention showed the strongest predictor, and additional predictors were the habit of encouraging patients to engage in physical activity and barriers that obstruct the encouragement of patients to engage in physical activity.

**Table 5:** Predictors of intention at T1 and behavior that encourages physical activity in cardiovascular patients at T2

*N = *278	Intention T1		Behavior T2	
	*r*	β	*r*	β
Intention			.44***	.311***
Attitude	.59***	.443***	.27***	
Perceived behavioral control	.41***	.137*	.28***	
Subjective norms	.45***	.201***	.25***	
Descriptive norm	.25***		.29***	
Moral norm	.14*		ns	
Habit	.27***		.30***	.163**
Barriers	-.38***		-.40***	-239***

R^2^		.41***		.29***

Regression,*** p < .001, ** p < .01, * p < .05;

r correlations;

β beta's

## Discussion

The study reported here explored healthcare professionals' intention to encourage physical activity among cardiovascular patients and the subsequent behavior of encouraging patients. We investigated the relative importance of social-cognitive variables derived from the Theory of Planned Behavior and other relevant social psychological theories in relation to healthcare professionals' intention and behavior, and the congruence between healthcare professionals' intention and behavior.

In this study, we demonstrated that social-cognitive variables are useful predictors of both healthcare professionals' intention to encourage physical activity in cardiovascular patients and their self-reported behavior of encouraging patients. Social-cognitive variables accounted for 29% of the variance in behavior, and 41% of the variance in intention. We found that behavior was predicted by high levels of intention, established habits of encouraging patients and low levels of barriers. Our finding that behavior is largely predicted by intention corresponds with previous studies including studies that focus specifically on the behavior of healthcare professionals [1,30,35,42,43].

Our results indicate that, among healthcare professionals, the intention to encourage physical activity among cardiovascular patients is strongly associated with attitude, subjective norms, and perceived behavioral control, and, to a lesser extent, descriptive and moral norms. That attitude, subjective norms and perceived behavioral control are significant predictors of intention is congruent with other research, including research focusing particularly on healthcare professionals' intention [1,5,29,30,34,44-46].

In a systematic review on the predictors of healthcare professionals' behavior, social-cognitive determinants predicted 35% of the variance in behavior and 59% of the variance in intention [30]. As in our study, a meta-analytic review showed that social-cognitive variables accounted for 27% of the variance in behavior and 39% of the variance in intention [34]. Previous studies have shown regression values of 36% for behavior and 42% to 66% for intention [29,44,46]. According to a meta-analysis and the above mentioned studies, the amount of variance in behavior explained in our study was smaller than the amount of variance in intention [42].

The social-cognitive determinants of healthcare professionals are consequently expected to be key objectives for cardiovascular morbidity-reducing interventions directed at increasing healthcare professionals' intention to encourage physical activity in cardiovascular patients and the behavior of encouraging patients. Our findings suggest that, in order to optimize healthcare professionals' behavior, strengthen both behavioral intention and habit are worthwhile, and reduce barriers.

In addition, our findings revealed that professionals with more positive attitudes, more positive subjective norms, and higher perceived behavioral control were more likely to have positive intentions to encourage physical activity in cardiovascular patients. Professionals perceived more advantages than disadvantages to encouraging cardiovascular patients to become physically active. Interventions should therefore endeavor to highlight and clarify the advantages of encouraging patients while providing explanations for the disadvantages. Healthcare professionals should get convinced that being physically active is important for patients with cardiovascular risk factors.

Also, subjective norms was in our study, found to be associated with intention, thus suggesting that professionals who felt that encouraging patients is expected and also done by other healthcare professionals had more positive intentions. Interventions should therefore be directed at seeking positive social support from other healthcare professionals, and learn to communicate the importance of encouraging patients to engage in physical activity with other healthcare professionals.

In our study, perceived behavioral control was found to be associated with the intention to encourage physical activity in cardiovascular patients, thus suggesting that professionals felt competent to perform the behavior and that encouraging patients was perceived as relatively easy to do. Interventions should support the feeling of competence and teach skills necessary to handle difficult situations encouraging patients to become or stay physically active.

The habit of encouraging patients can also contribute to behavior of encouraging patients and should therefore be consolidated via environmental interventions that strengthen such habits [40,47]. Our results suggest that investing in the development of the skills necessary to overcome barriers is worthwhile.

In addition to exploring the role of social-cognitive variables in the prediction of intention and behavior, we explored the relationships between intention and behavior. Do professionals with positive intentions actually encourage cardiovascular patients to become physically active? How many professionals have low intentions and do not engage in the behavior of encouraging patients to engage in physical activity? In our study, only 40% of the healthcare professionals had positive intentions and engaged in the self-reported behavior of encouraging patients and about 32% had low intentions and did not encourage cardiovascular patients to engage in physical activity. About 10% of the professionals had positive intentions but did not display the behavior of encouraging patients and about 17% had low intentions but nonetheless engaged in the behavior of encouraging patients to become physically active. This lack of congruence between high versus low intention and the behavior of encouraging patients versus not encouraging patients is an important input for interventions.

Interventions should be directed at strengthening the intention to encourage physical activity and the corresponding encouraging behavior for healthcare professionals. The social-cognitive determinants of intentions and behaviors may be changed in a more positive direction by planned interventions, deciding which determinants need to be changed, which need to be reinforced, and which need to be introduced [41,48]. To translate our results into an intervention for healthcare professionals aiming at increasing a positive intention towards encouragement of patients with cardiovascular risk factors and increasing their encouragement behavior, we will use Intervention Mapping. Intervention mapping provide a framework to built a systematically planned theory- and evidence-based intervention, highly applicable to the desired population and to improve the quality of interventions [41,48]. Intervention Mapping places specific emphasis on the input from social-cognitive determinants in the beginning of the development phases of an intervention.

Although the survey was specifically designed for the target population (nurses and physical therapists) with specific behavior (encouraging physical activity) for a specific group of patients (at risk for cardiovascular disease), it seems plausible that the basics could be used for other research. One strength of the study reported here is its longitudinal design as this ruled out the possibility that behavior caused intention [42]. The study's sample size is an additional strength. A possible limitation is that all of the professionals included in this study were recruited from the same institute and this may limit generalization of the outcomes. The measure of behavior is self reported and probably not very explicitly operationalized, this is also a possible limitation.

## Conclusions

In order to prevent and limit cardiovascular risk via the improvement of risk behavior in patients, it is imperative that we understand healthcare professionals' intention to encourage physical activity in cardiovascular patients and subsequent behavior of encouraging patients. A major implication of this study is that healthcare professionals' intention to promote physical activity in cardiovascular patients and self-reported behavior of encouraging patients can be predicted by social-cognitive determinants. This implies that efforts to change behavior and strengthen the intention-behavior relationship of healthcare professionals to encourage physical activity in cardiovascular patients can optimize the cardiovascular risk profile of patients.

## Competing interests

The authors declare that they have no competing interests.

## Authors' contributions

BS was involved in the study design, collected and analyzed the data, and took the lead in writing the manuscript. BS and GK designed the survey, contributed to the interpretation of the data, and provided feedback on the manuscript. All authors approved the final manuscript.

The original Dutch questionnaire is available for future research projects.

## Pre-publication history

The pre-publication history for this paper can be accessed here:

http://www.biomedcentral.com/1471-2458/11/246/prepub
